# Factors Influencing Support for National Health Insurance among Patients Attending Specialist Clinics in Malaysia

**DOI:** 10.5539/gjhs.v5n5p1

**Published:** 2013-05-14

**Authors:** Yasmin Almualm, Sharifa Ezat Alkaff, Syed Aljunid, Syed Sagoff Alsagoff

**Affiliations:** 1 Department of Community Health, Faculty of Medicine, National University of Malaysia, Kuala Lumpur, Malaysia; 2 United Nations University, International Institute for Global Health (UNU-IIGH), Kuala Lumpur, Malaysia; 3 Faculty of Law, National University of Malaysia, Kuala Lumpur, Malaysia

**Keywords:** National Health Insurance (NHI), National Health Financing Authority (NHFA), Social Health Insurance (SHI), support, knowledge, IQR: Interquartile range, MOH: Ministry of Health, NHI: National Health Insurance, NHFA: National Health Financing Authority, OR: Odds Ratio, RM: Ringgit Malaysia, USD: United States Dollar, SPSS: Statistical Package for Social Sciences, SHI: Social Health Insurance, WHO: World Health Organization

## Abstract

This study was carried out to determine the level of support towards the proposed National Health Insurance scheme among Malaysian patients attending specialist clinics at the National University of Malaysia Medical centre and its influencing factors. The cross sectional study was carried out from July-October 2012. 260 patients were selected using multistage sampling method. 71.2% of respondents supported the proposed National Health insurance scheme. 61.4% of respondents are willing to pay up to RM240 per year to join the National Health Insurance and 76.6% of respondents are of the view that enrolment in NHI should be made compulsory. Knowledge had a positive influence on respondent's support towards National Health Insurance. National Health Insurance when implemented in Malaysia can be used to raise funds for health care financing, increase access to health services and achieve the desired health status. More efforts should be taken to promote the scheme and educate the public in order to achieve higher support towards the proposed National Health Insurance. The cost to enroll in NHI as well as services to be included under the scheme should be duly considered.

## 1. Introduction

The *Malaysian Health Care System* is a success story in comparison to countries of the same socio-economic status (Marican & Yon, 2002). There has been an outstanding progress in healthcare system over the years and the country enjoys a comprehensive range of health care services. The government's commitment to provide affordable and high quality care is ensured by abundance of public health services which range from curative and rehabilitative care as well as health promotion and disease prevention (Ministry of Health [MOH], 2010). The health status in Malaysia is relatively good and the country is on target in achieving the Millennium Development Goals (World Health organization [WHO], 2010). Healthcare services in Malaysia are provided at all government facilities and are highly subsidized for all population regardless of their level of income, However, there are many issues that threaten the performance and sustainability of the country's health care system against a background of rising health expenditure (MOH, 2010). There is a gradual rise in demand and expectation from the public, high cost of medical care, changing disease patterns, demographic shifts and high out of pocket expenditure in Malaysia which is estimated at 40% of the total expenditure on health care services (MOH, Health Expenditure Report). These prevailing factors have triggered an inevitable need for health care reform. The World Health Organization has passed a resolution in 2005 that supports a strategy to gain more resources for health to increase access to health care for the poor and provide quality healthcare services in all its member states. Financial limitation is one of the major barriers of access to health care services for the poor in many societies. Moving away from out of pocket payment for health services at the time of use to prepayment through health insurance is an important step towards avoiding financial hardship associated with paying for health care services (Acharaya et al., 2012). In order to reduce the overdependence on direct payment governments must promote the prepayment mechanism. In fact, only when there is a prepayment that pools risk across the population then universal coverage can be achieved (WHO, 2010). The Ministry of Health have been entrusted to establish a *non-profit* entity known as National Health Financing Authority (NHFA) mandated to set-up and manage National Health Insurance (hereinafter referred to as NHI) otherwise known as *Social Health Insurance Scheme* (Marican & Yon, 2002) whereby all citizens will contribute a monthly premium while the government concurrently contributes a larger premium to the majority of the account holders. The amount of contribution by citizens to join the scheme and health services covered under the NHI is yet to be announced. Social Health Insurance (hereinafter referred to as SHI) is one of the mechanism used to raise and pool funds for health financing (Doetinchem, Carrin, & Evans, 2009). Contributions by the public towards social health insurance are based on their income level and not their health status (Carrin, 2002). The objective of Social Health Insurance is to provide healthcare that avoids large out of pocket spending, better utilization of health services and Improve health status (Acharaya et al., 2010). The principle behind Social Health Insurance is gaining popularity in developing countries and is one of two main options towards achieving universal health coverage. However the development of such mechanism depends on the country's socioeconomic background and requires a strong political will and high administrative capabilities (Carrin et al., 2009). The country's economic structure and development influence how many people can be covered and how fast SHI can expand toward achieving universal coverage (Hsiao & Shaw, 2007). Success of Social Health Insurance also requires the support of the population and the governing body of the country (Smith et al., 2008). Major changes in the health sector are frequently met with resistance from interest groups. The population sometimes are sceptical about promises of improvement, thus political will as well as a rational evaluation of the problems and opportunities are required before introducing major health financing reform (Doetinchem et al., 2009). Twenty seven countries have established the principle of universal coverage via Social Health Insurance (Carrin & James, 2005). Many low to middle countries are currently in the process of implementing Social Health Insurance (Ghosh & Mondal, 2011). This study was carried out to determine the level of support among the public towards the proposed National Health Insurance scheme in Malaysia and its influencing factors.

## 2. Methodology

This is a cross sectional study carried out from July until October 2012 at the specialist clinics at the National University of Malaysia Medical Centre. A total of 260 patients were selected using multistage sampling method. The National University of Malaysia Medical Centre was selected using convenience sampling. The Specialist clinics were selected by convenience sampling, the clinics include: Surgical, Medical, Obstetric and Gynecology and Dermatology. Malaysian patients who are 18 years old and above attending the selected specialist clinics were selected by systemic random sampling method. Systemic random sampling carried out by selecting every third name on the list of patients who were scheduled to attend the clinic on that day for follow up. Each respondent was personally interviewed using a newly developed structured questionnaire that has been validated by experts in health management and health economics. The questionnaire consisted of five sections addressing factors under study: (a) socio-economic and socio-demographic; (b) knowledge about the proposed National Health Insurance, knowledge was explored using a cumulative scale of which the median score was used to divide respondents into two groups, good knowledge and poor knowledge; (c) ownership of private health insurance, this section included questions regarding private health insurance ownership, yearly premiums, coverage and satisfaction with insurance provider; (d) monthly premiums to join NHI was explored using a likert scale of starting with not willing to contribute and ending with amount up to RM100, current exchange rate 1 USD= RM3.04; and (e) support for the proposed National Health Insurance Scheme, support was measured using a likert scale, respondents were given five options to express their support (or lack of it) towards NHI and these options are; strongly support, support, neutral, reject and strongly reject, then it was categorized into two groups which are support NHI and do not support NHI. Written and verbal consent were taken from each respondent. Statistical Package of Social Science (SPSS) software, version 19.0 was used in analyzing the data.

## 3. Results

A total of 260 respondents participated in this study. 24 respondents were excluded from the study due to incomplete questionnaire. The total sample size that was analyzed is 236. The response rate was 90.76%. From the 236 respondents there were 147 (62.3%) male respondents and 89 (37.7%) female respondents. 77.1% were Malays followed by 12.3% Chinese and 7.6% Indians. Respondent's age was set at minimum of 18 years and above Median age is 36.5 IQR 22. Median income of respondents is RM4 000 and range RM2 408.75, respondents were divided into lower income group and higher income group (using the median as a cut-off point). With regards to education, 55.4% of respondents had an education level of STPM (equivalent to diploma) or higher and the remaining 44.6% had an education level of SPM (secondary school) or lower (PMR is Primary school). 39.4% of respondents are working in the public sector while 29.7% are working in the private sector and 16.5% are self-employed. The remaining 14.4% are unemployed. 90.3% of respondents live in urban area.

### 3.1 Private Health Insurance

Of the total number of respondent (n=236), 127 (53.81%) respondent have an insurance plan that provides health coverage, out of those 127 respondent with an insurance plan, 117 (49.6%) respondents have private health insurance and 56 (23.7%) respondents have other types of insurance that provide health coverage (46 respondent had both, private health insurance and another insurance plan that provides health coverage). Of the 127 respondents who have an insurance plan that provides health coverage, 75 (59.1%) were satisfied with their insurance provider while 52 (40.9%) were not satisfied with their insurance provider. The median annual premium for private health insurance is recorded at RM1000, and IQR RM1400. Among respondents who have private health insurance (n=117), 59.8% of them chose NHI over private health insurance when asked about their preferred health insurance.

### 3.2 Knowledge on NHI

Knowledge on the proposed NHI was measured using cumulative scale of 1-10. The scale comprises of ten questions associated with knowledge. The median score for the study population is 6 and IQR of 2. The study finds that 73 (30.9%) respondents had good knowledge on the proposed NHI while 163 (69.1%) respondents had poor knowledge. Respondents were asked to choose one option they thought of as the most important factor that could hinder the success of National Health Insurance scheme in Malaysia. The factor that was chosen the most by respondents is poor knowledge. Results are shown in Table 1.

**Table 1:** Respondent's choice of factors that could hinder the success of NHI in Malaysia (n=228)

Factors that could hinder the success of NHI	Frequency (Percentage)
Premiums are high	82 (36)
Referral is not easy	24 (10.5)
Poor knowledge of scheme	99 (43.4)
Limitation of benefit package	23 (10.1)

### 3.3 Premiums Respondents Are Willing to Pay to Join NHI

It refers to the amount of money in Ringgit Malaysia respondent is willing to pay monthly per person to join the National Health Insurance scheme. It was explored using a scale of 1-6. Starting with RM0 (not willing to contribute) and ending with amount up to RM100. The amount that respondents are willing to contribute to NHI was explored using a fixed amount of money instead of a percentage of income. This is because a percentage of income is difficult to ascertain for those who do not have a fixed income. Also, having a high income does not automatically make a person willing to contribute more. This study intended to find out the amount of money that a respondent is willing to pay per month to join NHI. The study finds that, 16 (6.8%) respondents were not willing to contribute to the National Health Insurance. 135 (57.2%) respondents are willing to contribute up to RM20 per month to National Health Insurance. 32 (13.6%) respondents are willing to contribute up to RM60 per month. 31 (13.1%) are willing to pay up to RM40 per month and the remaining 18 (7.6%) are willing to contribute up to RM100 per month to NHI. The amount that respondents are willing to contribute monthly to join NHI is shown in Table 2.

**Table 2:** Amount of money respondents are willing to contribute per month to join NHI

Amount they are willing to contribute Monthly to join NHI	Frequency (percentage)
Not willing to pay	16(6.8)
Up to RM 20	135 (57.2)
Up to RM 40	31 (13.1)
Up to RM 60	32 (13.6)
Up to RM 80	4 (1.7)
Up to RM 100	18 (7.6)
Total	236 (100)

192 (91.9%) respondents are of the view that rehabilitation services should be covered under the National Health and 211 (98.1%) respondents thought that treatments for chronic illnesses should be covered under the National Health Insurance scheme.

### 3.4 Support towards the Proposed NHI in Malaysia

Support towards the proposed National Health Insurance was measured using a likert scale of 1-5, then results were categorized into two groups which are support NHI and do not support NHI. Of the 236 respondent who participated in this study, 168 (71.2%) respondents supported the proposed NHI scheme while 68 (28.8%) did not support. Results are shown in Table 3.

**Table 3:** Frequency and percentage of acceptance towards NHI among respondents

Support towards NHI	Frequency (Percentage)
Support	168 (71.2)
Don’t support	68 (28.8)
Total (n)	236 (100.0)

### 3.5 Factors Influencing Public's Support towards the Proposed NHI

Chi square was used to test the relationship between the dependant and independent factors. p value of less than 0.05 is considered significant. The dependent variable for this study is respondents support towards NHI.

Association between Socio-economic and demographic factors and respondents support for NHI

None of the factors were found to have significant association with respondents support for NHI. Chi square results for socio-economic and demographic factors are shown in Table 4.

**Table 4:** Association between socio-economic and demographic factors and respondents support towards NHI

	Support NHI Frequency (percentage)	Do not support NHI Frequency(percentage)	Chi square	p value
Age (years)			0.331	0.565
≤ 36	82 (69.5)	36 (30.5)		
> 36	86 (72.9)	32(27.1)		
Household monthly income			0.009	0.925
≤ RM4 000	89(72.4)	34(27.6)		
>RM4 000	61(71.8)	24(28.2)		
Education			0.108	0.743
Low	72 (69.9)	31(30.1)		
High	92(71.9)	36(28.1)		
Gender			0.488	0.485
Male	107(72.8)	40(27.2)		
Female	61(68.5)	28(31.5)		
Employed			1.310	0.252
Yes	141(69.8)	61(30.2)		
No	27 (79.4)	7(20.6)		
Chronic illness			0.156	0.693
Yes	43(72.9)	16(27.1)		
No	120(70.2)	51(29.8)		
Marital status			0.489	0.484
Yes	121(69.9)	52(30.1)		
No	47(74.6)	16(25.4)		
Number of Children			0.005	0.942
≤3	81(71.7)	32(28.3)		
>3	39(72.2)	15(27.8)		
Area			0.443	0.506
Urban	153(71.8)	60(28.2)		
Rural	15(65.2)	8(34.8)		

### 3.6 Association between Having an Existing Private Health Insurance and Respondents’ Support for NHI

Of the total number of respondent (n=236), 117 respondent have private health insurance and 56 respondent have other types of insurance that provide health coverage. There was no significance association between having private health insurance plan and respondents acceptance towards NHI. There was no significant association between having another type of insurance that provide health coverage and respondent's support for NHI. Results are shown in Table 5.

**Table 5:** Association between having private health insurance and respondent's support towards NHI and between having other types of insurance that provides health coverage and acceptance towards NHI

	Support NHI Frequency (percentage)	Do not support NHI Frequency (percentage)	Chi square	p value
Having PHI*			1.139	0.286
Yes	87(74.4)	30(25.6)		
No	81(68.1)	38(31.9)		
Having other insurance			3.010	0.083
Yes	45 (80.4)	11(19.6)		
No	123(68.3)	57(31.7)		

* PHI: Private Health Insurance

Respondent's Satisfaction with insurance provider was also tested with respondent's support towards NHI. A total of 145 respondents have insurance plan that provides health coverage (private health insurance or other type of insurance). Respondent's satisfaction with their insurance provider had no significant association with respondent's support for NHI. Preferred choice of health insurance among respondents with private health insurance plan was also tested with respondent's support for NHI. Among respondents who have private health insurance plan (n=117) there was a significant association between their preferred choice of health insurance and support for NHI. Respondents with private health insurance plan who chose NHI over private health insurance as their preferred type of insurance are more supportive for NHI compared to those who chose private health insurance as their preferred type of insurance, p was significant at 0.010. Results are shown in Table 6.

**Table 6:** Association between satisfaction with insurance provider and respondent's support towards NHI and association between preferred choice of health insurance among respondents with private health insurance and support towards NHI

	Support NHI Frequency (percentage)	Do not support NHI Frequency (percentage)	Chi square	p value
Satisfaction with insurance provider (n=127)			1.806	0.179
Yes	61(81.3)	14(18.7)		
No	37(71.2)	15(28.8)		
Preferred insurance among respondent with PHI *(n=117)			6.600	0.010**
NHI	58(82.9)	12(17.1)		
PHI	29(61.7)	18(38.3)		

* PHI: Private Health Insurance

** p value significant at < 0.05

### 3.7 Association between Premiums Respondents’ Are Willing to Pay to Join NHI and Respondents’ Support towards NHI

The amount of money in Ringgit Malaysia respondent is willing to pay monthly per person to join the National Health Insurance scheme was assessed using a likert scale of 1-5. Starting with amount of RM1-RM20 and ending with amount up to RM100. Respondents were divided into two groups. There was no significant association between amount they are willing to pay monthly to join NHI and respondent's support for NHI. Results are shown in Table 7.

**Table 7:** Association between monthly premiums for NHI and respondent's support towards NHI

Monthly premiums	Support NHI Frequency (percentage)	Do not support NHI Frequency (percentage)	Chi square	p value
≤RM 40	122(73.5)	44(26.5)	1.404	0.236
RM41-RM100	44(81.5)	10(18.5)		

### 3.8 Association between Knowledge about NHI and Respondents’ Support towards NHI

Respondent's knowledge on the proposed NHI in Malaysia was assessed using a score of 1-10. The median score for knowledge in this study is 6. A score of 1-6 is considered poor knowledge. A score of 7-10 is considered good knowledge. There was a significant association between knowledge and respondent's support for NHI. Respondents who have good knowledge on NHI are more supportive towards NHI compared to respondents who have poor knowledge on NHI, p value was significant at 0.001. Results are shown in Table 8.

**Table 8:** Association between respondent's knowledge on NHI and respondent's support towards NHI (n=236)

Knowledge on NHI	Support NHI Frequency (percentage)	Do not support NHI Frequency(percentage)	Chi square	p value
Good knowledge	63(86.3)	10(13.7)	11.772	0.001*
Poor knowledge	105(64.4)	58(35.6)		

* p value significant at < 0.05

### 3.9 Predicting Effects of all the Factors on Public Support towards NHI

In order to determine which of the independent variables had the most predicting effect on respondent's support for NHI, binary logistic regression was carried out using the Enter model. Socio-economic and demographic factors had no predicting effect on respondent's support towards NHI. Premiums respondents are willing to pay to join NHI, having private health insurance and choice of health insurance among respondents did not have an predicting effect on respondent's support for NHI. Knowledge had a predicting effect on respondent's support for NHI with p value of 0.009 and odds ratio (OR) of 3.398 and a 95.0% confidence interval (CI) of 1.360-8.485. This can be interpreted as: a respondent with good knowledge on NHI is 3.398 times likely to support NHI compared to a respondent with poor knowledge. Results of binary logistic regression are shown in Table 9.

**Table 9:** Binary logistic regression of respondent's support towards MHI, the enter model

Variables	B	S.E.	Wald	df	Sig.	Exp(B)	95% C.I. for EXP(B)	

							Lower	Upper
Chose NHI over private health insurance	.676	.380	3.155	1	.076	1.966	.932	4.143
Lower income group	.251	.440	.326	1	.568	1.286	.543	3.046
Willing to pay lower amount	-.124	.468	.070	1	.791	.883	.353	2.211
Have private health insurance	.385	.396	.944	1	.331	1.470	.676	3.196
Employed	.943	.816	1.335	1	.248	2.567	.519	12.703
Lower education	.021	.418	.003	1	.960	1.021	.450	2.317
Male	.488	.383	1.624	1	.203	1.629	.769	3.451
Have chronic illness	-.138	.447	.095	1	.758	.871	.362	2.093
Married	-.642	.510	1.586	1	.208	.526	.194	1.429
Good knowledge	1.223	.467	6.859	1	.009	3.398	1.360	8.485
Constant	.415	.724	.329	1	.566	1.514		

## 4. Discussion

### 4.1 Support towards the Proposed National Health Insurance Scheme in Malaysia

Prior to implementing major health reform, it is important to assess the support of the public. Particularly in Malaysia, where health care services are highly subsidized. Many factors determine the level of support towards government policies. This study intended to find out the level of support towards NHI in Malaysia and how the factors under study influenced public support. In a society with high solidarity, individuals are more willing to support each other. This is important because a financial protection requires cross-subsidization between rich and poor and between low risk and high risk people (Carrin & James, 2005). Studies carried out in countries that are in the process of implementing Social Health Insurance had mixed results. A study on support for Social Health Insurance in South Africa shows that 53 per cent of respondents supported Social Health Insurance if scheme members were assured preferential treatment (Smith et al., 2008). A study on demand for Health Insurance among the urban poor in India finds out that only 35 per cent of household heads interviewed were willing to join the scheme (Ghosh & Mondal, 2011). While a study in Namibia on demand for Health Insurance finds out that 87 per cent of the uninsured respondents are willing to join the proposed Health Insurance scheme (Gustafsson-Wright, Asfaw, & Van der Gaag, 2009). In this study, support towards National Health Insurance among Malaysian attending specialist clinics at the UKMMC is high. This result is consistent with another study carried out among farmers in Selangor, Malaysia (Aizuddin et al., 2011).

### 4.2 Association between Socio-Economic and Demographic Factors and Respondents’ Support towards National Health Insurance Scheme

The country's economic structure and development influence how many people can be covered under SHI (Hsiao & Shaw, 2007). Per capita income influences how much people can actually contribute towards SHI. Higher per capita income increases the ability of the people to contribute to SHI (Carrin & James, 2005). The size of the formal and the informal sector in the country also matters. When the informal sector is big, SHI is likely to face administrative difficulties in setting the amount of contribution (since their income is difficult to ascertain) and also how these contributions are being collected. Similar studies (Gustafsson-Wright et al., 2009; Aizuddin et al., 2011; Ghosh & Mondal, 2011; Jehu-Appiah et al., 2011) have cited low socioeconomic status as a significant factor for lack of support and poor enrolment in social health insurance. In this study, none of the socio-economic and demographic factors were found to have significant association with respondents support towards NHI. But, it should be mentioned that the studies that were referred to here, in exception to South Africa, were carried out in low income countries such as India and African countries. The economic status is different from Malaysia which is a high middle income country. Also the target population in those studies is different. While they focused on poor and informal sector, this study was carried out in an urban area and majority of respondents worked in the formal sector. To the researchers best knowledge, there are no studies carried out in Malaysia that asses the support of the formal sector towards the proposed NHI. In a previous study carried out in Malaysia (Aizuddin et al., 2011) on willingness to join and contribute to National Healthcare Financing scheme, it focused on the farmers in Selangor in which age, education level and per capita income of respondents influenced willingness to pay for National Healthcare Financing scheme But 87.3% of respondent in that study were more than 40 years old (Mean 56.9, SD 12.6), 74.5% of respondents had low education and the median monthly household income was RM755, IQR RM739 which make it different from this study population.

### 4.3 Association between Having Private Health Insurance and Respondents’ Support towards NHI

In private health insurance the risk-pool is relatively small making it difficult to cross-subsidize between different risk-groups (Sekhri, Savedoff, & Thripathi, 2005). Premiums are based on the health risk of the individual and not his income, to the extent that providers actually have incentives to select whom they want to insure. Raising premiums for high-risk individuals or even declining to ensure sick individuals. Private health insurance is profit oriented, there is a preferential treatment to those who can afford and this leads to inequality. This is different from the concept of SHI in which contribution are based on level of income and not the health status of an individual. There was no significance association between having private health insurance plan and respondents support towards NHI. There was no significant association between having other type of insurance that provide health coverage and respondent's support towards NHI. This is in contrast to a study carried out in South Africa (Smith et al., 2008) in which respondent with private health insurance had low support towards Social Health Insurance. Respondent's satisfaction with their insurance provider had no significant association with respondent's support towards NHI. Preferred choice of health insurance among respondents with private health insurance plan had significant association with respondent's support towards NHI (p= 0.010). 59.8% of respondents who have private health insurance chose NHI over private health insurance. This is not surprising since in SHI their premium will be based on their income and not their health risk, so this probably indicates their desire for a health insurance plan that is of a lower cost then what they are currently paying.

### 4.4 Association between the Cost to Join NHI and Respondents’ Support towards NHI

How much a person is willing to pay to join a health insurance scheme is not necessarily a reflection of his income. A study on health insurance in Namibia shows that rich people are willing to pay more than double that of those who are poor but poor respondents were willing to pay more than 11 percent of their income compared to rich who are willing to pay only 1.22 percent of their income (Jehu-Appiah et al., 2011). A study by Aizuddin et al. (2011) addressing the informal sector in Selangor finds that 76.5% of respondent are willing to pay between RM1.00 to RM5.99 for outpatient charges in public health facilities. This study shows that on a monthly basis, 135 (57.2%) respondents are willing to contribute up to RM20 to National Health Insurance. Also, 85.9% of respondents thought that contributions should be calculated based on number of family members. Thus, a family of four is willing to contribute RM20 per month per person will be paying a total of RM960 a year to NHI. This is approximate to the median annual premium for private health insurance reported in this study which is RM1000. There was no significant association between amount they are willing to pay monthly to NHI and respondent's support.

### 4.5 Association between Knowledge on NHI and Respondents’ Support towards NHI

Education and promotion is important before implementing major policies. It gives the public an understanding of why such policies were introduced, how such policies intend to serve the public and what are the potential benefits. In this study, there was a significant association between knowledge and respondent's support towards NHI (p=0.001). Knowledge positively influenced respondent's support towards NHI. This is consistent with similar studies (Asenso-Okyere, Osei-Akoto, Anum, & Appih, 1997; De Allegri, Sanon, & Sauerborn, 2006; Basaza, Criel, & Van der Stuyft, 2008; Ghosh & Mondal, 2011). Also, when respondents were asked to choose one option they thought of as the most important factor that could hinder the success of National Health Insurance scheme in Malaysia, the factor that was chosen the most by respondents is poor knowledge. This indicates the need to promote NHI among the Malaysian public in order to provide a clear understanding of how such a scheme can be beneficial at an individual level as well as national level. Regression analysis also shows that knowledge is a predicting factor on respondent's support towards NHI with p 0.009 and OR of 3.398 and a 95.0% CI of 1.360-8.485. In other words: Respondent with good knowledge on NHI is 3.4 times likely to support NHI than a respondent with poor knowledge.

## 5. Conclusion

The study shows high support towards the proposed NHI among Malaysian patients attending specialist clinics at The National University of Malaysia Medical Centre. NHI when implemented can raise funds for health care financing, increase access to health services and achieve the desired health status. This is in tandem with WHO resolution to achieve universal health coverage as well as the concept behind 1 Care for 1 Malaysia. More efforts could be taken to promote the scheme and educate the public through media, campaigns and seminars. The cost to enroll in NHI as well as services to be included under the scheme should be duly considered.

### Limitations

Limited resources did not permit a larger sample size. When implementing major health reforms it is important to assess public's support and its influencing factors on a large scale. Future studies should also include examining other factors that may influence public's support towards the future NHI in Malaysia such as quality of life and satisfaction with health care services.

### Recommendations

The study indicates that 71.2% of respondents supported NHI. More can be done to increase the support among the public. Raising awareness and promoting NHI through media, campaigns and seminars. The cost to enroll in NHI as well as services to be included under the scheme should be duly considered. More studies should be carried out in Malaysia to assess the support of the public towards NHI and what are the factors that determine their support. This can help policy makers in understanding the needs and aspirations of the very people they intend to serve.
